# Effects of early postnatal gastric and colonic microbiota transplantation on piglet gut health

**DOI:** 10.1186/s40104-023-00954-w

**Published:** 2023-12-25

**Authors:** Christina Larsen, Simone Margaard Offersen, Anders Brunse, Mattia Pirolo, Soumya Kanti Kar, Luca Guadabassi, Thomas Thymann

**Affiliations:** 1 Department of Veterinary and Animal Science, University of Copenhagen, Dyrlægevej 68, 1870 Frederiksberg C, Denmark; 2 Animal Nutrition, Wageningen Livestock Research, Wageningen University & Research, 1 De Elst, 6708 Wageningen, The Netherlands

**Keywords:** Colonic content filtrate transplantation, Colonic microbiota transplantation, Gastric microbiota transplantation, Gut microbiota, Mucosa, Neonatal, Post-weaning diarrhea

## Abstract

**Background:**

Diarrhea is a major cause of reduced growth and mortality in piglets during the suckling and weaning periods and poses a major threat to the global pig industry. Diarrhea and gut dysbiosis may in part be prevented via improved early postnatal microbial colonization of the gut. To secure better postnatal gut colonization, we hypothesized that transplantation of colonic or gastric content from healthy donors to newborn recipients would prevent diarrhea in the recipients in the post-weaning period. Our objective was to examine the impact of transplanting colonic or gastric content on health and growth parameters and paraclinical parameters in recipient single-housed piglets exposed to a weaning transition and challenged with enterotoxigenic *Escherichia coli* (ETEC).

**Methods:**

Seventy-two 1-day-old piglets were randomized to four groups: colonic microbiota transplantation (CMT, *n* = 18), colonic content filtrate transplantation (CcFT, *n* = 18), gastric microbiota transplantation (GMT, *n* = 18), or saline (CON, *n* = 18). Inoculations were given on d 2 and 3 of life, and all piglets were milk-fed until weaning (d 20) and shortly after challenged with ETEC (d 24). We assessed growth, diarrhea prevalence, ETEC concentration, organ weight, blood parameters, small intestinal morphology and histology, gut mucosal function, and microbiota composition and diversity.

**Results:**

Episodes of diarrhea were seen in all groups during both the milk- and the solid-feeding phase, possibly due to stress associated with single housing. However, CcFT showed lower diarrhea prevalence on d 27, 28, and 29 compared to CON (all *P* < 0.05). CcFT also showed a lower ETEC prevalence on d 27 (*P* < 0.05). CMT showed a higher alpha diversity and a difference in beta diversity compared to CON (*P* < 0.05). Growth and other paraclinical endpoints were similar across groups.

**Conclusion:**

In conclusion, only CcFT reduced ETEC-related post-weaning diarrhea. However, the protective effect was marginal, suggesting that higher doses, more effective modalities of administration, longer treatment periods, and better donor quality should be explored by future research to optimize the protective effects of transplantation.

**Supplementary Information:**

The online version contains supplementary material available at 10.1186/s40104-023-00954-w.

## Background

The porcine intestine contains trillions of microbes that are important for gut homeostasis and host health [1]. After farrowing, the intestine is colonized with microbes from the environment and the sow’s vagina, feces, and skin [2]. Microbes play an essential role in nutrient absorption, metabolism, development of the immune system, differentiation of the intestinal epithelium, and maintenance of the intestinal mucosal barrier [3, 4]. Hence, microbial colonization becomes an important determinant of gut stability and robustness in later life, including the susceptibility to post-weaning diarrhea (PWD) [5]. The abrupt transition from a highly digestible milk diet to a more complex solid diet at weaning is associated with a drop in lactobacilli and a higher risk of opportunistic pathogen overgrowth [6–8]. This microbial perturbation can lead to a loss in bacterial diversity [5, 9] and results in susceptibility to enteric pathogens such as enterotoxigenic *Escherichia coli* (ETEC) [10]. Although PWD can be effectively controlled with antibiotics, their use may select for antimicrobial resistance and result in perturbation of the gut microbiome (i.e., dysbiosis) [11]. Alternative treatment strategies to replace antibiotics use are therefore required.

One possible alternative is fecal microbiota transplantation (FMT), which is the transfer of a fecal suspension from a healthy donor to a recipient to reshape the intestinal microbiota [12]. Previously, FMT has been used to re-establish a normal gut microbiome in humans to cure *Clostridioides difficile* infections [13, 14]. In recent years, FMT has been shown to decrease the occurrence of PWD and improve growth rate by improving gut microbiota composition and diversity [15–19]. However, other studies have shown negative effects on gut microbiota, growth performance, absorptive capacity, and intestinal health, possibly due to inappropriate donor-recipient matches in terms of age or animal breed [20, 21]. Donor-recipient gut microbiota compatibility may be important for both safety and efficacy [22], which may also depend on the clinical condition of the recipient at the time of transplantation.

Fecal transplantation inherently carries a risk of transferring pathogenic microbes from donor to recipient. Filtration of feces (FFT) to achieve a suspension free of bacteria, fungi, and parasites may offer a means to improve safety and has shown promising safety and efficacy profiles for managing human *Clostridioides difficile* enterocolitis [23] and piglet bowel inflammation [24]. Considering that oral administration is the most feasible way to perform microbiota transplantation in pigs, an alternative inoculum could be the gastric content of a healthy donor, i.e., gastric microbiota transplantation (GMT). The stomach microbiota at 8–14 days of age is dominated by lactobacilli [25], and several studies found a positive effect of probiotics containing lactobacilli on intestinal health [26, 27].

Based on this background, we hypothesized that transplantation of colonic microbiota (CMT), colonic content filtrate (CcFT), and GMT from healthy suckling piglet donors to newborn recipients on the first day of life would improve growth and reduce ETEC diarrhea through modulation of the gut microbiota. Colonic material was used to ensure enough material and reflects the bacterial composition in feces [28]. We addressed this hypothesis using single-housed piglets exposed to a weaning transition and challenged with ETEC. This study provides new insights into different microbiota transplantation modalities as alternatives to antibiotics for PWD prophylaxis in pigs.

## Materials and methods

### Animals housing and management

Seventy-two 1-day-old colostrum-immunized piglets (Danbred (Duroc × Danish Landrace × Yorkshire)) from 21 sows in parities 2–5 (1–5 piglets from each sow with equal gender distribution) were purchased from a commercial sow herd (Holbæk, Denmark). Upon arrival at the animal facility, the piglets were initially housed together in groups of three until they had learned to drink milk from a trough. Thereafter, all piglets were housed individually in cages (90 cm × 74 cm). Cages were cleaned once per day and equipped with enrichment material, heating lamps, and ad libitum access to water. Troughs and water supply were cleaned twice per day to ensure hygiene. The room temperature was kept constant at 26 °C from d 2 until the end of the experiment (d 29). All piglets received a subcutaneous injection of iron dextran complex (Uniferon, 1 mL/pig, Unitron a/s, Kolding, Denmark) for prevention of anemia and a single oral treatment with toltrazuril (Baycoxine Vet. 0.4 mL/kg, Elanco ApS, Ballerup, Denmark) for prevention of coccidiosis on d 3 and 4 of life, respectively.

### Experimental diet and feeding

During the initial 20 days of the experiment (i.e., 21 days of age), all piglets received a milk replacer diet consisting of a mixture of bovine milk enriched with whey protein (Bulk Powder Performance, Bulk, Essex, United Kingdom and WPI/WPC 90, Arla Foods Ingredients P/S, Viby, DK) and whey permeate (Variolac 836, Arla Foods Ingredients P/S, Viby, DK (Table S1)). Daily, all piglets received 180 mL per metabolic body weight (kg^0.75^) from d 1 to 3, 210 mL from d 4 to 7, 222 mL from d 8 to 14, and 240 mL from d 15 to 19. Milk bolus feeding was provided every second hour, i.e., 12 meals per day, using an automatic milk feeding system (Big Dutchman, Vejen, Denmark). From d 20 to 21 the piglets were weaned and received 100 g of solid creep feed (Table S2), and from d 21 onwards, they were given ad libitum access to solid feed.

### Experimental design

**Fig. 1:**
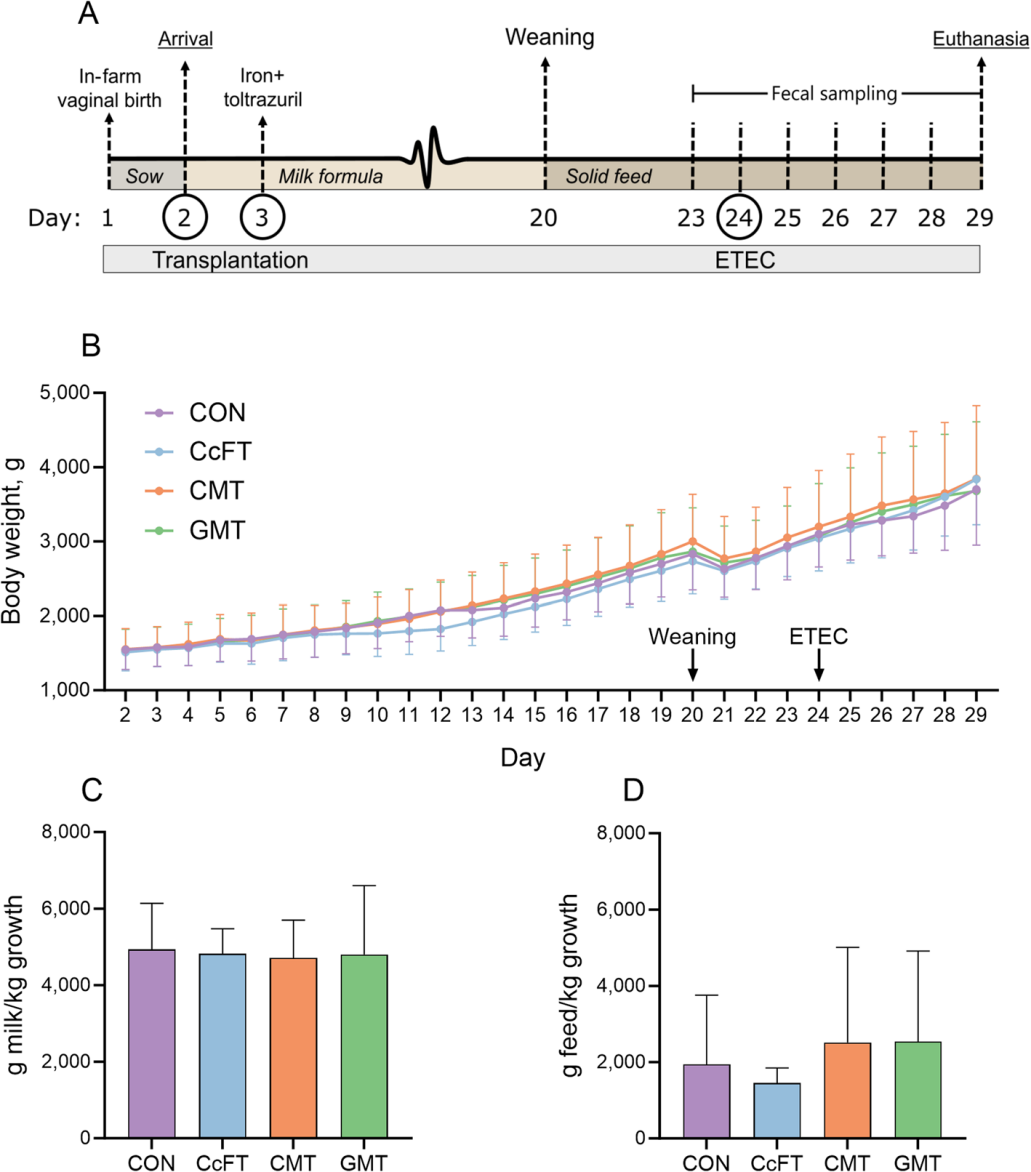
**A** Study design. **B** Growth curve based on daily body weight of pigs from d 1 to 29. The pigs received either colonic content filtrate transplantation (CcFT, *n* = 16), colonic microbiota transplantation (CMT, *n* = 15), gastric microbiota transplantation (GMT, *n* = 15), or saline (CON, *n* = 16). **C** Feed conversion ratio of pigs from d 2 to 20. **D** Feed conversion ratio of pigs from d 21 to 29. Data are expressed as mean ± SD

The animals were stratified according to sex and body weight and randomly allocated to one of four groups receiving oral administrations of colonic microbiota transplantation (CMT, *n* = 18), colonic content filtrate transplantation (CcFT, *n* = 18), gastric microbiota transplantation (GMT, *n* = 18) or saline (CON, *n* = 18). Participants of this study were blinded to the treatment groups. The study was conducted over two experimental rounds with nine piglets per treatment group in each round. Figure 1A illustrates the study design.

### Inoculum preparation and administration of transplants

Colon luminal and gastric contents were collected from fifteen 11-day-old healthy suckling piglets from the same farm as the recipient piglets. All donor piglets appeared to be clinically healthy without signs of diarrhea, displayed normal body weight relative to their age and were raised without use of antibiotics or pharmacological concentrations (2,000–3,000 mg/kg) of zinc oxide in the diet. After euthanization, the colon and gastric luminal content respectively was pooled across the animals, homogenized and diluted 1:1 with 20% sterile glycerol, and stored at −80 °C until use. Before inoculation, colon and gastric content were further diluted 1:3 in sterile saline to a working concentration of 0.17 g content/mL (CMT and GMT). Furthermore, half of the CMT was centrifuged at 5,000 × *g*, 4 °C for 30 min, and the supernatant was filtered through a 0.45-µm syringe filter (Filtropur S, PES, Sarstedt, Germany) after which it was ready to administer (CcFT). The inoculum was administered via a feeding tube into the stomach on the day of arrival (d 2) and d 3. The CMT, CcFT, and GMT groups received 6 mL of working solution per treatment (corresponding to 1 g of original material diluted in 3.3% glycerol) and the CON group received equivalent volumes of sterile saline with 3.3% glycerol. The donor material was screened for the following pathogens: ETEC, rotavirus, *Lawsonia, Salmonella*, and *B. pilosicoli* (Kjellerup laboratory, Landbrug & Fødevarer F.m.b.A. SEGES Laboratory for Swine diseases) and only donors free of these pathogens were used in the final inoculum.

### ETEC challenge

On d 24, all piglets received an oral single dose of ET10 (O149:H10, F4ac, STb, LT) gelatine capsule containing 10^5^ CFU/capsule. The procedure of the inoculum has been previously described in Rydal et al. [29]. The capsules were kept at −20 °C until use and the CFU was determined by cultivation after thawing.

### Clinical and performance endpoints

All piglets were weighed daily throughout the study (Bjerringbro vægte, model no. APM-60, Bjerringbro, Denmark). The feed conversion ratio (FCR) was measured as the weight of feed intake over a period divided by the weight gained during the period. From d 1 to 23, clinical and fecal scores were assessed visually once daily. From d 24 to 29 clinical score was assessed three times per day. The clinical status was scored according to a clinical system (1 = normal, 2 = mild symptoms, 3 = moderate symptoms, 4 = severe symptoms). The fecal score was recorded as either normal or diarrhoeic and daily prevalence in each group was calculated as (total cases of diarrhea on a specific day/piglet group size) × 100. Leftovers of both milk and solid feed were collected and weighed twice per day during the experiment. On d 23–28, stool samples were collected and kept at −80 °C for later ETEC quantification.

### Sample collection

All piglets were anesthetized on d 29 with an injection of a mix of zolazepam (25 g/mL, Virbac, Kolding, Denmark), tiletamine (25 g/mL, Virbac, Kolding, Denmark), ketamine (100 g/mL, MSD Animal Health, Copenhagen, Denmark), xylazine (20 mg/mL, ScanVet Animal Health A/S, Fredensborg, Denmark), and butorphanol (10 mg/mL, Biovet ApS, Fredensborg, Denmark). When full anesthesia was achieved, blood samples were drawn by cardiac puncture into heparinized vacutainers. Afterward, the piglets were euthanized with an intra-cardiac injection of sodium-pentobarbital (400 mg/mL, ScanVet Animal Health A/S, Fredensborg, Denmark). The spleen, liver, and kidney were harvested and weighed. The stomach and colon were weighed before and after it was emptied and rinsed with tap water. Furthermore, the small intestine (SI) was measured in length and weighed full and empty. Tissue from jejunum was taken for brush border enzyme activities, morphology, and histology. Colon luminal content was collected for 16S rRNA amplicon sequencing analysis and ETEC qPCR analysis, as described below.

### F4-ETEC quantification

ETEC concentration was estimated by quantitative PCR (qPCR) on 1:10 dilution of stool samples using primers F4-F 5′-CACTGGCAATTGCTGCATCT-3′ and F4-R 5′-ACCACCGATATCGACCGAAC-3′ [30] amplifying the *faeG* gene (F4ac). Real-time PCR assay was performed on the LightCycler 96 System (Roche Life Science, Copenhagen, Denmark) in 20 μL reactions with FastStart Essential DNA Green Master mix (Roche Life Science, Copenhagen, Denmark) with the additions of each primer at a concentration of 0.5 μmol/L. The cycling conditions were as follows: 2 min at 95 °C; 40 cycles of 15 s at 95 °C and 60 s at 60 °C; and a melt curve step from 60 to 95 °C. A qPCR standard curve was created with tenfold dilutions of ETEC strain ET10 ranging from 10^7^ to 10^1^ CFU/mL. The DNA from both diluted stool samples and ETEC culture of calibration curve was extracted with the boiling method. The number of target copies in each sample was then calculated using the equation: copy number = [10^(−1/S)^]^(I − Ct)^, where S is the slope of the log-linear part of the standard curve, I the intercept of the standard curve, and Ct is the cycle threshold of the sample. Copies were normalized for gram of feces and the limit of detection was set to 36 Ct, equal to ~ 100 F4ac copies/reaction. Daily ETEC prevalence within each group was calculated as (total cases of > 100 F4ac on a specific day/piglet group size) × 100.

### Health indices measures in blood 

Serum cytokine and chemokine concentrations (pg/mL) were measured using a ProcartaPlex Porcine kit (Affymetrix, eBIOscience, Vienna, Austria). Calibration curves from recombinant cytokine and chemokine standards were prepared for the 8-point standard dilution set with fourfold dilution steps in sterile PBS. The samples were measured using a Bio-Plex MagPix Multiplex Reader (Bio-Rad Laboratories Inc. by the Luminex Corporation, The Netherlands). The Bio-Plex Manager software’s five-parameter logistic curve fitting (5PL) method was used for raw data analysis and calculation of cytokine concentrations. Using the manufacturer’s protocol of commercial ELISA kits, we quantitatively measured the levels of three acute-phase proteins (APP) in blood serum: pig haptoglobin (Abcam, ab205091, Cambridge, United Kingdom), pig C-reactive proteins (CRP; Abcam, ab205089, Cambridge, United Kingdom), and pig major acute phase protein (MAP, ACUVET, Acuvet Biotech, Zaragoza, Spain).

Clinical biochemistry and hematology were measured in EDTA-stabilized (BD-Plymuth, PL6, 7BP, UK) blood. Upon centrifugation, plasma was isolated from EDTA-stabilized blood, and biochemical profiles were determined using an Advia 1800 chemistry system (Siemens Healthcare Diagnostics, Tarrytown, NY, USA).

### Intestinal histology and mucosal function

Histomorphology of the intestine and measurement of enzyme activity formalin-fixed tissue samples from distal jejunum were dehydrated in ethanol and embedded in paraffin, and stained with hematoxylin and eosin. Morphometric analysis of villus height (µm), crypt depth (µm), enterocyte height (µm), number of infiltrating epithelium lymphocytes per 100 enterocytes (IEL/100E), number of goblet cells per 100 enterocytes (goblet cells/100E) were done with Zen Blue 3.0 software at ALAB Weterynaria (Warsaw, Poland). Histopathological lesions (infiltration of the stromal mucosa, mucosal epithelium, brush border, intestinal blunting, cell detritus rich in eosinophils, eosin, edema of stromal mucosa, vessel dilation in stromal mucosa of villi, infiltration of submucosa, edema of submucosa, hyperplasia of enterocytes, cell detritus in the villi surface, number of mitoses in intestinal crypts, hyperemia, vacuolization of neurons) were assigned to a 5-point scale (0 = no pathological changes, 1 = minimal, 2 = mild, 3 = moderate and 4 = marked). The gut-associated lymphoid tissue evaluation included the number of lymphoid follicles visible in intestinal sections.

As a marker of gut mucosal function, we measured the activity of aminopeptidase N, aminopeptidase A, dipeptidyl IV, maltase, sucrase, and lactase in distal jejunum SI, using the assay as described in Sangild et al. [31].

### Intestinal microbiota 

Total DNA from recipient colon content samples and two replicates of each inoculum was extracted using the QIAamp UCP Pathogen MiniKit (QIAGEN, Copenhagen, Denmark), according to the manufacturer’s instructions, with the addition of a bead-beating step using the Pathogen Lysis tube S (QIAGEN, Copenhagen, Denmark). A blank extraction control was included in the DNA extraction protocol. Partial 16S rRNA gene sequences were amplified using the Quick-16S NGS Library Prep Kit (Zymo Research, CA, USA), which targets the V3-V4 region of the 16S rRNA gene, as previously described [32]. Negative and positive control (ZymoBIOMICS DNase/RNase Free Water and ZymoBIOMICS Microbial Community DNA Standard, respectively) were included in library preparation. Sequencing was performed on an Illumina MiSeq platform (2 × 300 bp paired-end reads) using the MiSeq Reagent Kit v3 (600 cycles; Illumina), according to manufacturer’s instructions. The 16S rRNA sequencing data have been submitted to the NCBI Sequence Read Archive (SRA) under BioProject PRJNA981444.

16S rRNA sequencing data were processed using DADA2 v1.14.1 [33] as implemented in R v4.2.1. Optimal filtering and trimming parameters were identified using FIGARO v3.0 [34]. A taxonomy table was assembled by assigning taxonomy to each amplicon sequence variant (ASV) using the Silva taxonomic database v.138.1 for DADA2 [35]. Potential contaminants were identified using control samples and removed using decontam v.1.12.0 [36]. Sequences matching mitochondria or chloroplast were also removed, along with any sequences not assigned to bacteria. A phyloseq object was constructed from the ASV and taxonomy tables using phyloseq v1.30.0 [37] for subsequent analysis.

To enumerate culturable aerobic bacteria, 100 μL of 10-fold dilutions of each inoculum were spotted in triplicates on blood agar plates. Colonies were counted after incubation at 37 °C for 24 h under aerobic conditions.

### Data calculations and statistical analysis

Data analysis was performed using software R (version 2022.02.1 + 461, R Foundation for Statistical Computing, Vienna, Austria), and illustrations were done in GraphPad Prism (Version 9.3.1 (471), GraphPad Software, La Jolla CA, USA). Repeated measurements over time for continuous variables (growth, feed intake, quantitative ETEC counts) were analyzed using linear mixed-effect models. The prevalence of diarrhea and ETEC on each day were analyzed with pairwise logistic regressions. Average daily gain (ADG), FCR, the first incidence of diarrhea, health indices (cytokines, chemokines, and APP), clinical biochemistry, hematology, relative organ weights, morphology, histopathology, and enzyme activity were analyzed using linear models. All models included the following fixed effects: treatment, sex, experimental round, and either birth weight (diarrhea and ETEC prevalence, FCR) or sacrificed weight (biochemistry, hematology, morphology, health indices, relative organ weight, and enzyme activity). Validation of the linear models was done by testing the normality and homoscedasticity of the residuals and fitted values. If data did not meet the assumptions, data were log-transformed or transformed by reciprocal to meet the criteria. Validated linear models were analyzed with an ANOVA on the treatment level followed by a Tukey post-hoc test. The ordinal histopathology data was analyzed using the non-parametric Kruskal–Wallis and Dunn's post hoc test analysis with Benjamin-Hochberg correction. Data are presented as means and standard deviations, except histological parameters which are presented as median and interquartile range (IQR). *P*-values below 0.10 were regarded as a tendency and *P*-values below 0.05 were regarded as statistically significant.

For 16S rRNA sequencing data, alpha-diversity (Shannon and Chao1) and beta-diversity (Bray–Curtis dissimilarity metric) indexes were calculated using R package vegan after rarefication with a depth of 90% of the minimum sample depth in the dataset. Multiple comparison of alpha-diversity indexes was performed using the Wilcoxon Rank Sum test and *P*-values were corrected for multiple comparisons using Holm’s correction. Beta-diversity was visualized using a principal coordinates analysis (PCoA) plot, and differences in beta-diversity were estimated by permutational multivariate analysis of variance (PERMANOVA) using the Adonis function. Differential abundance analysis between treatments and control was performed using DESeq2 and contrasts were corrected for multiple comparisons using the Benjamini-Hochberg’s correction. Only ASVs with adjusted *P*-values < 0.05 and estimated fold change > 5 were considered significantly differentially abundant and were visualized in a heatmap from R package complex-heatmap. Code for data analysis can be accessed in the Github repository (https://github.com/mpirolo/AVANT-WP1-FMT-trial).

## Results

### Survival and growth performance

Ten pigs distributed over the four groups (2 CON, 2 CcFT, 3 CMT, and 3 GMT) were euthanized during the study due to severe weight loss. Relative to CON, the three intervention groups (CcFT, CMT, and GMT) showed comparable growth curves during the study (Fig. 1B). Average daily gain was similar across groups in both the milk period (66.5 ± 3.47 g/d), the post-weaning period (90.8 ± 12.8 g/d), and the post-ETEC period (121 ± 20.3 g/d).

The overall milk and feed intake were similar in the three intervention groups compared to CON (Fig. S1), which were the same for the FCR in all periods (Fig. 1C and D).

### Diarrhea and ETEC 

**Fig. 2:**
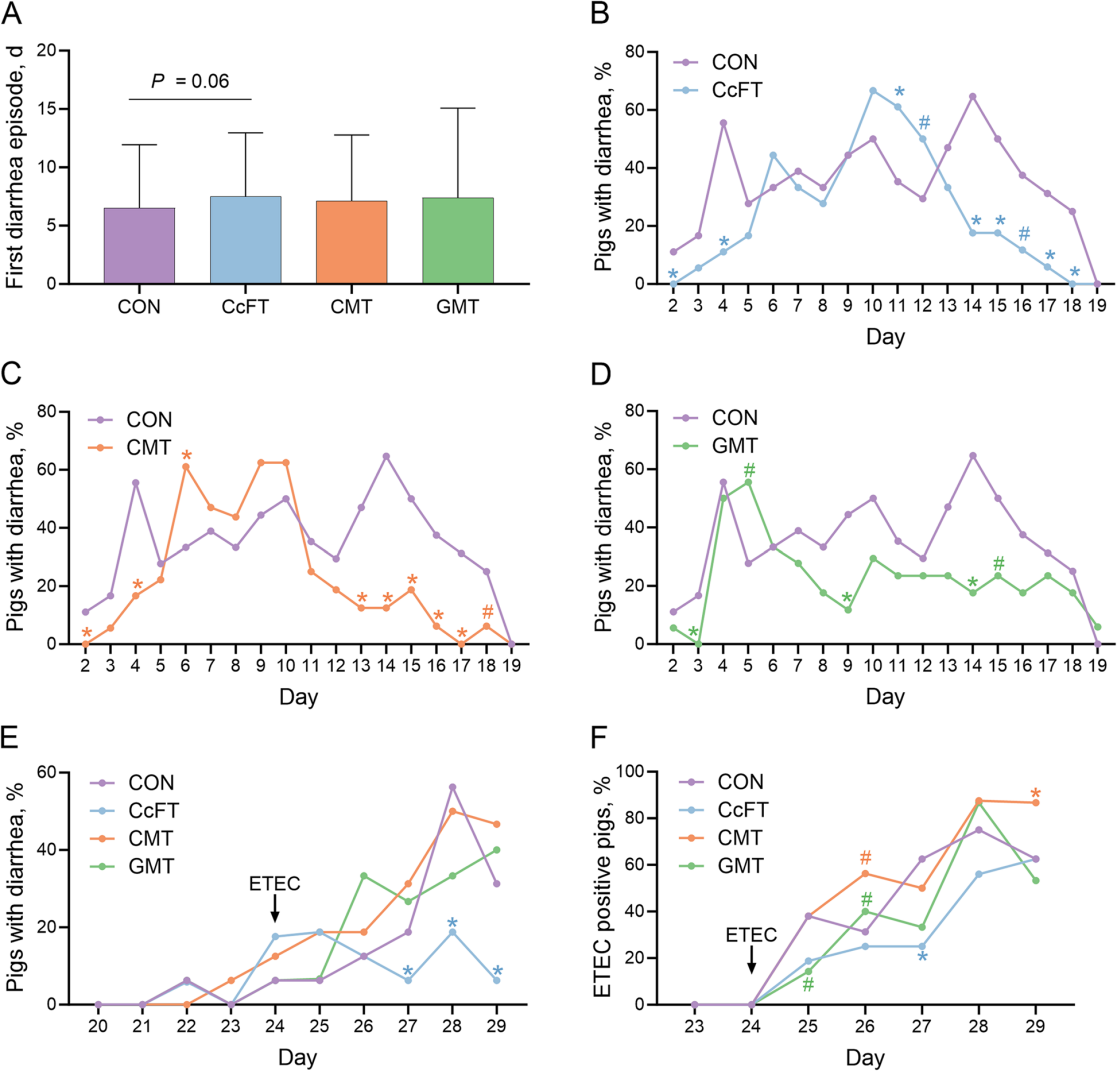
The pigs received either colonic content filtrate transplantation (CcFT, *n* = 16), colonic microbiota transplantation (CMT, *n* = 15), gastric microbiota transplantation (GMT, *n* = 15), or saline (CON, *n* = 16). **A** First time to diarrhea. **B** Daily diarrhea prevalence of pigs in CcFT vs. CON from d 2 to 19. **C** Daily diarrhea prevalence of pigs in CMT vs. CON from d 2 to 19. **D** Daily diarrhea prevalence of pigs in GMT vs. CON from d 2 to 19. **E** Daily diarrhea prevalence of pigs from d 20 to 29. **F** Daily ETEC prevalence by qPCR of pigs from d 23 to 29. Each treatment group was compared to CON with logistic regression models. Data are expressed as mean ± SD. ^*^P < 0.05, ^#^*P* = 0.06–0.10

During the milk period, a high prevalence of diarrhea was observed in all four groups. Notably, the onset of diarrhea was delayed by approximately 1 d in CcFT compared to CON (*P* = 0.06). CON had the first diarrhea episode on d 6.5 ± 5.43, whereas CcFT had the first episode of diarrhea on d 7.5 ± 5.5, (Fig. 2A). Furthermore, CcFT had a lower diarrhea prevalence than CON on d 2 (*P* = 0.004), 4 (*P* = 0.01), 14 (*P* = 0.0008) 15 (*P* = 0.03), 17 (*P* = 0.04), and 18 (*P* = 0.005). On the contrary, there was a significantly higher diarrhea prevalence in CcFT than in CON on d 11 (*P* = 0.04), 12 (*P* = 0.09), and 16 (*P* = 0.08) (Fig. 2B). CMT had a lower prevalence than CON on d 2 (*P* = 0.01), 4 (*P* = 0.01), 13 (*P* = 0.01), 14 (*P* = 0.0002), 16 (*P* = 0.01), and 17 (*P* = 0.003) but a higher prevalence than CON on d 6 (*P* = 0.04) (Fig. 2C). GMT had a lower diarrhea prevalence on d 3 (*P* = 0.03), 9 (*P* = 0.03), and 14 (*P* = 0.001) compared to CON (Fig. 2D). In the post-weaning period, CcFT was the only treatment group showing significantly lower diarrhea prevalence compared to CON on d 27 (*P* = 0.01), 28 (*P* = 0.02), and 29 (*P* = 0.04) (Fig. 2E).

On d 27, CcFT decreased the ETEC prevalence relatively by 60% compared to CON (*P* = 0.02). The ETEC prevalence in CMT was higher than in CON on d 26 (*P* = 0.08) and 29 (*P* = 0.04) by 37.5% and 27.9%, respectively. Whereas in GMT it was lower than in CON on d 25 (*P* = 0.10) and 27 (*P* = 0.08) by 62.4% and 46.7%, respectively (Fig. 2F). No significant trends in ETEC concentrations were observed in the four groups throughout the study (Fig. S2).

### Necropsy, histology, and mucosal enzyme activity

Organ weight, mucosal morphology, histopathology, mucosal enzyme activity, and all blood parameters were largely similar between the groups as shown in Tables S3, S4, S5, S6, S7, S8, and S9.

### Gut microbiota composition and diversity

**Fig. 3:**
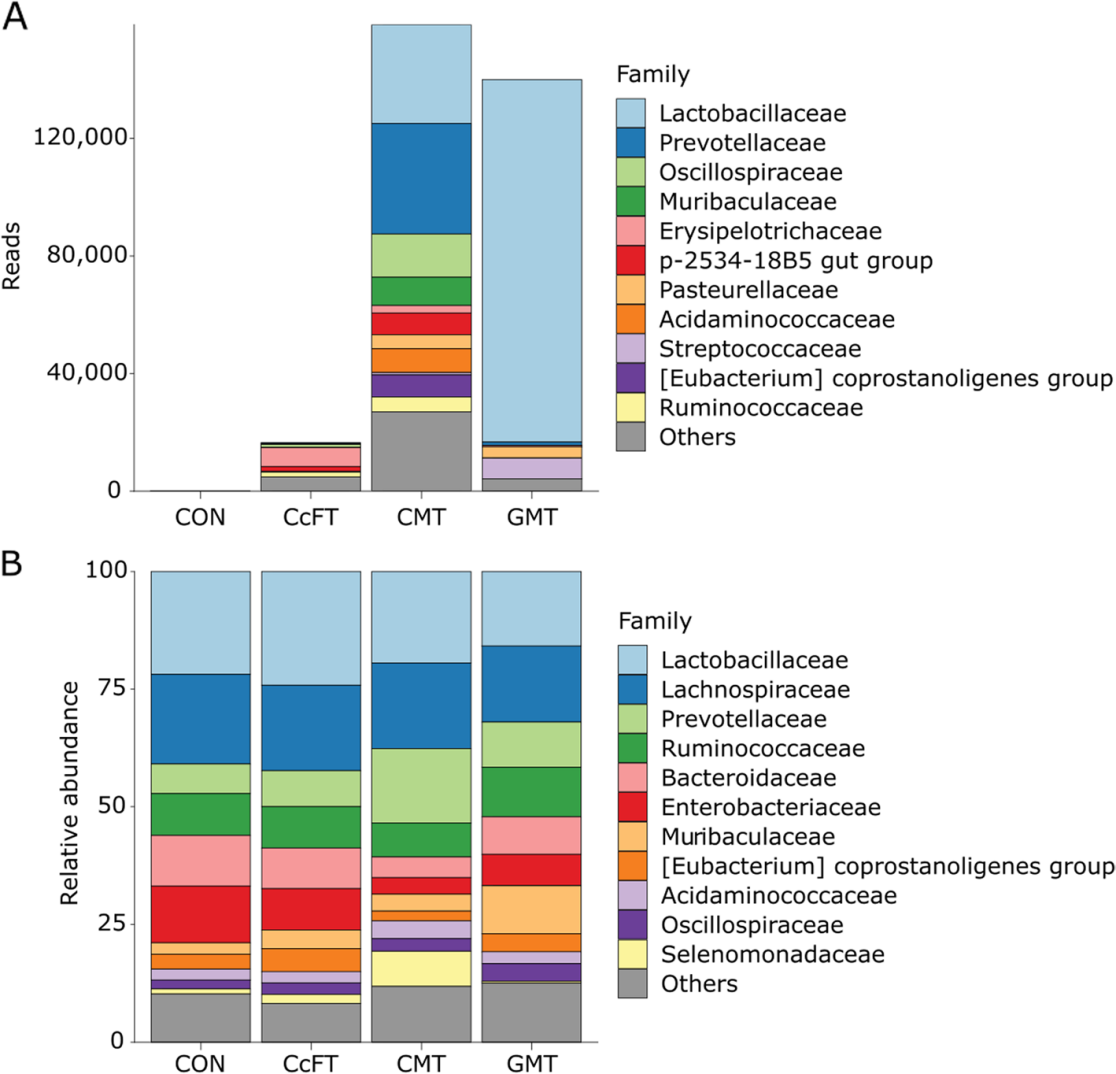
**A** Composition of bacteria (family level) in each inoculum (two replicates per inoculum). The inoculum were either colonic content filtrate transplantation (CcFT), colonic microbiota transplantation (CMT), gastric microbiota transplantation (GMT), or saline (CON). The top 10 most abundant taxa are displayed after filtering reads with an abundance > 100 among at least 2 samples. **B** Relative microbial abundance (family level) in recipient pigs on d 29. The pigs either received colonic content filtrate transplantation (CcFT, *n* = 16), colonic microbiota transplantation (CMT, *n* = 15), gastric microbiota transplantation (GMT, *n* = 15), or saline (CON, *n* = 16)

Figure 3 shows the bacterial composition at the family and genus levels of each inoculum. The core microbiome of CMT inoculum was formed by members of Lactobacillaceae, Prevotellaceae, and Oscillospiraceae, accounting for over 50% of reads. The genera *Lactobacillus* and *HT002* of Lactobacillaceae dominated the GMT inoculum, accounting for 61.2% and 22.1% of reads, respectively. Read counts in the CcFT inoculum (average 18,040 reads in 239 ASVs) were reduced compared to CMT (average 179,959 reads in 1,069 ASVs). Within CcFT inoculum samples, a single ASV assigned to an unclassified member of Erysipelotrichaceae accounted for 35.6% of reads. Bacteria were virtually absent in CON inoculum samples, which showed a mean reads count per sample of 32 and 7 ASVs. Aerobically culturable bacteria enumeration confirmed 16S rRNA results. The number of viable cell decreased from 3.6 × 10^7^ CFU/mL in CMT inculum to 5.6 × 10^2^ CFU/mL in CcFT inoculum. The GMT inoculum showed a viable bacterial cell count of 6.1 × 10^4^ CFU/mL, while no aerobic bacteria were detected in the CON inoculum.

**Fig. 4:**
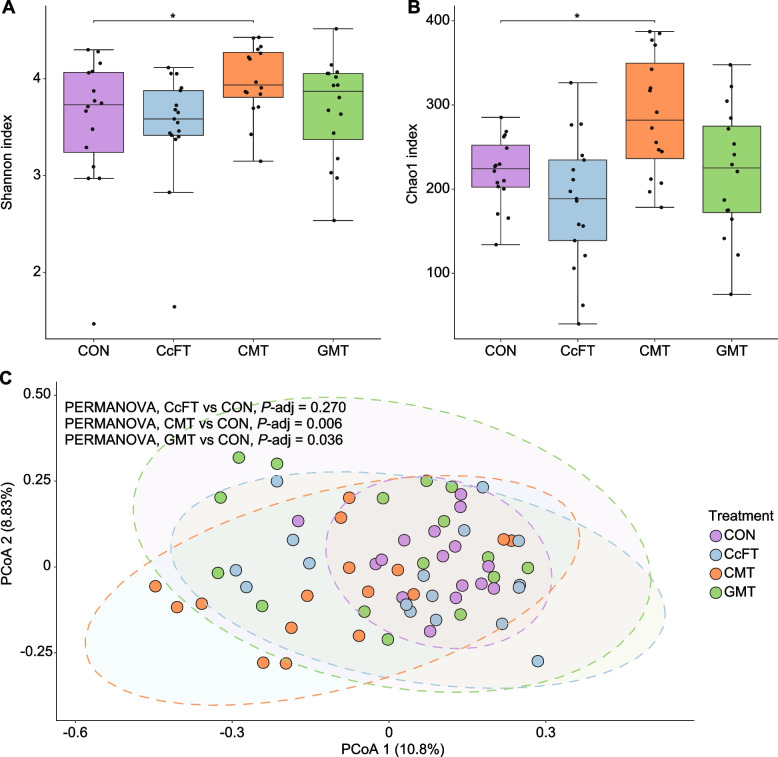
Comparison of alpha- and beta-diversity indexes of colon content from pigs receiving colonic content filtrate transplantation (CcFT, *n* = 16), colonic microbiota transplantation (CMT, *n* = 15), gastric microbiota transplantation (GMT, *n* = 15) or saline (CON, *n* = 16). **A** and **B** Box-plot of α-diversity calculated with Shannon (**A**) and Chao1 (**B**) indexes. **C** Two-dimensional principal coordinates analysis (PCoA) plot based on the Bray–Curtis dissimilarity matrix. Sample clustering of the CMT and GMT sample was significantly different from CON samples (PERMANOVAP < 0.05). ^*^P < 0.05

Figure 4 shows the bacterial diversity in recipient piglets. Compared to CON, treatment with CMT was accompanied by a significant increase in alpha diversity (Wilcoxon Rank Sum test, adjusted *P*-value < 0.05), expressed by both Chao1 and Shannon indexes (Fig. 4A and B). Conversely, there was no difference in alpha diversity between CcFT or GMT relative to CON (Fig. 4A and B). Community analysis based on Bray–Curtis dissimilarity matrix showed a different microbiota composition between samples collected from CMT or GMT and CON (PERMANOVA, adjusted *P*-value < 0.05) (Fig. 4C). Conversely, no significant changes in community composition were observed between CcFT and CON (PERMANOVA, adjusted *P*-value = 0.27) (Fig. 4C).

**Fig. 5:**
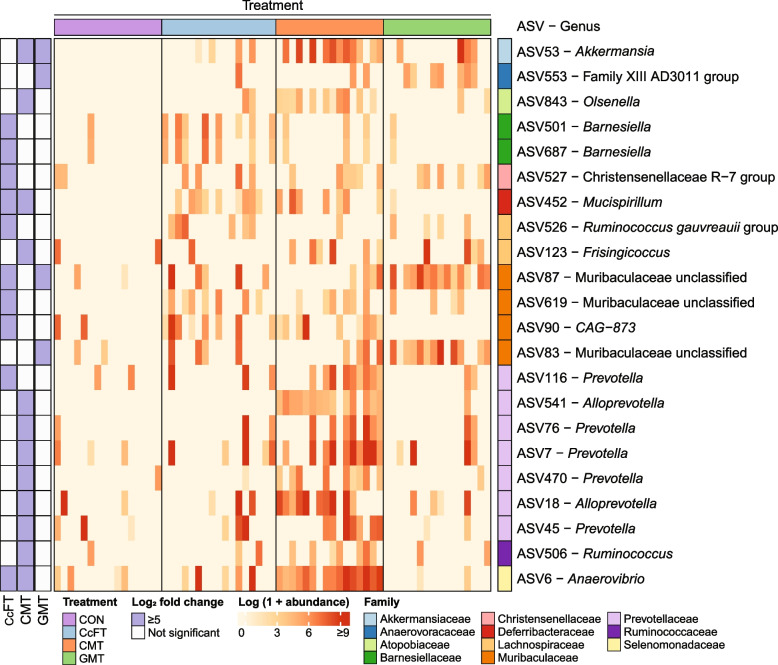
Differentially abundant amplicon sequence variants (ASVs) between pigs receiving colonic content filtrate transplantation (CcFT, *n* = 16), colonic microbiota transplantation (CMT, *n* = 15), gastric microbiota transplantation (GMT, *n* = 15) and saline (CON, *n* = 16). Only ASVs with adjusted *P*-values < 0.05 and estimated fold change > 5 were considered significantly differentially abundant and included in the plot

DESeq2 was used to identify ASVs differentially abundant between treatment groups and controls. The analysis identified 22 ASVs that were significantly associated (Wald test, adjusted *P*-value < 0.05, and estimated fold change > 5) with treatments compared to CON, including 12, 10, and 4 ASVs in CMT, CcFT, and GMT, respectively (Fig. 5). Four ASVs were more abundant in two intervention groups (Fig. 5). A significant increase in members of *Prevotellaceae* was observed in CMT samples compared to CON, including 5 ASVs assigned to *Prevotella* and 2 ASVs assigned to *Alloprevotella*. ASVs assigned to members of the Muribaculaceae family were significantly more abundant in both CcFT (*n* = 3 ASVs) and GMT (*n* = 2 ASVs) compared to CON. In CcFT samples, a higher relative abundance of the genus *Barneisella* (*n* = 2 ASVs) was observed compared to CON.

## Discussion

Immediately after birth, the gastrointestinal tract starts to become colonized with bacteria, viruses, bacteriophages, fungi, and parasites [24]. While the profile of microorganisms fluctuates substantially in early life, this exact period may also represent a window of opportunity for interventions with probiotics [38, 39]. This has drawn major attention not the least within human neonatology where inoculation with lactobacilli or bifidobacteria has become common practice in many hospital units [40]. However, the evidence for gut-protective effects of single or multi-strain early postnatal probiotics supplementation is not clear in human neonatology [40]. From the notion that gut-protective effects may require a much wider consortium of microorganisms, we used the approach of transplanting material from the stomach or the colon from healthy donor animals to neonatal pigs. The transplant was administered within the first day after birth based on previous studies indicating that this represents an optimal window to influence intestinal colonization. We found that early oral CcFT, but not CMT or GMT, reduced PWD occurrence and ETEC prevalence. These positive effects were however restricted to a few specific days and the other clinical paraclinical endpoints did not significantly differ from the control group.

CcFT primarily consists of bacterial debris, proteins, DNA, metabolites, and viruses [24]. The concept of using colonic content filtrates is derived from previous studies that have shown promising therapeutic effects in adult human patients suffering from *Clostridium difficile* overgrowth [23] and prophylactic effects against necrotizing enterocolitis in neonatal pigs [24]. While early postnatal colonization with a transplant may have acute or short-term beneficial effects on the recipient, it was not known if early postnatal transplantation could have more protracted effects that last beyond the weaning transition.

The development of the bacterial and viral microbiome following colonization with a transplant from a healthy donor remains to be fully understood. The positive effects observed in the CcFT group may be attributable to the presence of bacteriophages and bacterial metabolites, and possibly few bacteria remaining after filtration with a 0.45-µm filter. Whether the CcFT inoculum contains ETEC-specific bacteriophages are unknown. However, CcFT comprises a diverse range of bacteriophages that may alter the gut bacteria and protect the host from bacterial invasion [41, 42]. Additionally, alterations in the bacteriophage community have been linked with neonatal diarrhea in piglets [43]. Metabolites are found to affect intestinal epithelial cells by acting as an energy source for proliferation of stem cells as well as goblet cells and mucin secretion. Therefore, the metabolites may contribute to maintaining the host physiology [44] and thereby exert a positive effect on PWD. We cannot exclude that the remaining CcFT bacteria can have an effect. However, this is unlikely due to the low CFU count of 5.6 × 10^2^ CFU/mL.

Both studies in pigs and humans have demonstrated a positive effect of FMT in early life by improving the barrier function of the intestine, likely due to increased bacterial diversity and changes in microbiota composition [15, 45]. Transplantation of gastric matter has to the best of our knowledge, not been described before in pigs. Although access to gastric content is not feasible without sacrificing the donor animal, we decided to investigate whether this inoculum rich in *Lactobacillus* could perform better than colonic content as oral supplementation in early life. *Lactobacillus casei* has previously been shown to improve growth performance and immunity and decrease diarrhea rates [26]. However, we did not observe any preventive effect of GMT on PWD or ETEC prevalence. The only effect was a shift in beta diversity compared to the CON group.

Based on the analysis of the microbiota composition of colon samples collected on d 29, all three interventions led to higher inter-individual bacterial heterogeneity compared to controls. CMT led to changes in beta diversity accompanied by a cocomitant increase in alpha diversity (Fig. 4C). In particular, CMT promoted the growth of beneficial bacteria, such as various members of Prevotellaceae, especially *Prevotella*, which is a dominant genus in the gastrointestinal tract of pigs [46] and has been associated with positive outcomes in pig production [47]. The changes in microbiota composition were only marginal in the CcFT and GMT. To increase the resolution necessary to appreciate these changes, a longitudinal study on pig microbiota development after transplantation should be carried out, possibly coupled with shotgun metagenomic sequencing.

We observed frequent episodes of diarrhea in all groups during the milk-feeding period. Due to the absence of the most common pathogens in the donor material and the occurrence of diarrhea in both the CON group and the experimental group, it is more likely that the diarrhea episodes were a result of the stress of single housing and shift from sow milk to milk replacer. Previous studies have demonstrated that such a transition can impact the integrity of the small intestinal barrier and raise the likelihood of experiencing diarrhea [48]. Notably, the overall high prevalence of spontaneous diarrhea among all groups may have masked the treatment effects and should therefore be regarded as a limitation of the study.

In contrast to previous findings, we did not observed a decrease in growth performance among the groups experiencing higher episodes of diarrhea. The piglets were restricted in their access to milk formula due to the risk of diarrhea, based on earlier experience from other studies at our research facilities [48]. Therefore, a lower growth rate in our piglets was expected compared to sow-reared piglets under conventional settings, which corresponds with the findings in Amdi et al. [49]. However, no differences in growth was observed between the three intervention groups and CON. Hu et al. [15] found that oral FMT administered for a long period (d 1–11) increased average daily gain using a total dose of ~ 0.8 g/pig compared to the 2 g/pig in our study. This indicates that the number of treatments, rather than the fecal dose, may be too low in our study, thereby limiting the effectiveness of the interventions.

16S rRNA gene sequencing of the inoculums showed that when pooled donor colon content was processed through centrifugation and filtration, there was a notable decrease in the number of bacterial taxa and culturable aerobic bacteria. This is indicated by the number of reads and viable cell count on blood agar, although a qPCR quantification of the 16S-region would be needed to fully compare the total bacterial load between inoculums. As free DNA is present in CcFT, there is a possibility that cell-free 16S regions were amplified in the CcFT inoculum samples. Within this inoculum, more than one-third of reads belonged to a single ASV assigned to an unclassified member of Erysipelotrichaceae family, which has been previously associated with the gastrointestinal tract of pigs [50, 51]. Considering the small size (0.2–0.4 µm diameter) of the most representative species (*Erysipelothrix rhusiopathiae*) in this family [52] it is plausible that these bacteria were able to pass through the filters used for preparation of the CcFT inoculum (0.45 µm diameter).

We only observed minor changes in the biochemistry profile on d 29, indicating no effect on liver and kidney function. Our results showed that the systemic cytokines and chemokines were largely unaffected and no differences were observed in the intervention groups. In addition, neither the ETEC challenge nor the intervention approaches used in this study induced an APP response of haptoglobin, CRP, and MAP, which is generally triggered as a result of infection by pathogens [53]. Collectively, these results indicate the absence of any pathological signatures at the systemic level in the experimental groups 5 d after ETEC challenge.

We assessed morphology and histopathology parameters of the SI as well as indices of mucosal function as activity of six different digestive enzymes. All values were similar between CON and the three intervention groups. As these data represent a cross-section at the time of tissue collection, we cannot exclude that there were transient differences in association with diarrhea episodes, but that all groups have converged toward similar levels at the time of tissue collection.

Although all piglets were challenged with ETEC, not all piglets displaying symptoms of PWD were effectively colonized by ETEC. This could be due to the relatively low inoculum used in our study (10^5^ CFU/mL). Rydal et al. [54] used an ETEC challenge to newly weaned piglets equal to 5 × 10^9^ CFU, whereas Jansman et al. [55] gave an oral inoculation to 7-day-old piglets equal to 1 × 10^9^ CFU/mL and observed ETEC-diarrhea. To clarify why the ETEC prevalence did not correlate with the occurrence of diarrhea, it would have been useful to know the MUC4 or CHCF1 genotype of the piglets before the start of the study [29]. Due to logistical issues, this was not possible. This observation, however, could also indicate that some episodes of the PWD that occurred during the experiment were not caused by ETEC.

Despite its merits, the present study has its limitations. The rationale behind the choice of relatively young (11-day-old) donors was that the microbiome is more similar between donors and recipients and therefore easier to establish in the recipient animals. However, this was not necessarily the best choice since a recent study has shown that older donors have a better effect on growth of the recipient piglets [56]. As sow-to-piglet transfer of microbiota at farrowing is considered beneficial, maternal feces could be used instead to improve the colonization of the gut and lower PWD as we observed in a previous study [57]. Another questionable choice was the use of colonic content instead of feces for microbiota transplantation, which requires sacrifice of the donors. This decision was made to enhance collection of a sufficient amount of material. In fact, although feces can be collected without sacrificing the donor animal, this approach is time-consuming and difficult to implement in practice when using young donor animals. Even though colon content and feces have similar bacterial compositions [28], the effects after transplantation may deviate. Qi et al. [58] observed differential effects of inoculation of a colonic and fecal microbiome in young piglets, which highlights that the use of microbiota transplantation in animal production requires careful selection and evaluation of the donor material. Finally, routes of administration other than intragastrical inoculation could be considered to limit the loss of active microbes in the acidic stomach. Rectal administration can be used, though only possible in smaller volumes. However, since ETEC colonizes the SI [59], rectal administration may be less effectfull against this pathogen.

## Conclusion

Intragastric transplantation of filtrates of colonic content from 11-day-old suckling piglets had a modest effect on preventing PWD and reducing ETEC prevalence, whereas transplants of intact colonic and gastric content were not effective. The mechanisms for beneficial effects of CcFT over CMT and GMT remain elusive as the majority of paraclinical endpoints, including bacterial profiles of colon luminal content, were largely similar between the groups. Focused research is needed to optimize dosage, mode of administration, and donor selection, as well as to determine the individual effects of various components of filtrated transplants, including small bacteria, bacteriophages, and metabolites.

### Supplementary Information

**Additional file 1: Fig. S1. **Feed intake.** Fig. S2. **ETEC prevalence.** Table S1. **Ingredients and calculated nutrient composition of the milk replacer provided to the piglets. **Table S2. **Ingredients and calculated nutrient composition of weaner diet. **Table S3.** Relative organ dimensions of piglets euthanized on d 29. **Table S4. **Small intestine morphology of piglets on d 29. **Table S5. **Small intestine histopathology of piglets on d 29. **Table S6. **Gut brush border enzymes of piglets on d 29. **Table S7. **Hematological parameters of piglets on d 29. **Table S8. **Biochemistry parameters of piglets on d 29. **Table S9. **Health indices in blood serum of piglets on d 29.

## Data Availability

The analyzed data from this study are available from the corresponding author on request.

## Abbreviations

ADG: Average daily gain
ASV: Amplicon sequence variant
CcFT: Colonic content filtrate transplantation
CON: Control
CRP: C-reactive proteins
ETEC: Enterotoxigenic *Escherichia coli*
FCR: Feed conversion ratio
FFT: Filtration of feces
FMT: Fecal microbiota transplantation
GMT: Gastric microbiota transplantation
IEL/100E: Infiltrating epithelium lymphocytes per 100 enterocytes
IQR: Interquartile range
MAP: Major acute phase protein
PCoA: Principal coordinates analysis
PWD: Post-weaning diarrhea
qPCR: Quantitative PCR
SI: Small intestine
SRA: Sequence Read Archive
