# Supplementary material for: Effects of early postnatal gastric and colonic microbiota transplantation on piglet gut health
Source: J Anim Sci Biotechnol. 2023 Dec 25;14:158. doi: 10.1186/s40104-023-00954-w (PMC10749501; doi:10.1186/s40104-023-00954-w)
Supplement: Supplementary file 1 — Additional file 1: Fig. S1. Feed intake. Fig. S2. ETEC prevalence. Table S1. Ingredients and calculated nutrient composition of the milk replacer provided to the piglets. Table S2. Ingredients and calculated nutrient composition of weaner diet. Table S3. Relative organ dimensions of piglets euthanized on d 29. Table S4. Small intestine morphology of piglets on d 29. Table S5. Small intestine histopathology of piglets on d 29. Table S6. Gut brush border enzymes of piglets on d 29. Table S7. Hematological parameters of piglets on d 29. Table S8. Biochemistry parameters of piglets on d 29. Table S9. Health indices in blood serum of piglets on d 29. [file 40104_2023_954_MOESM1_ESM.docx]

**
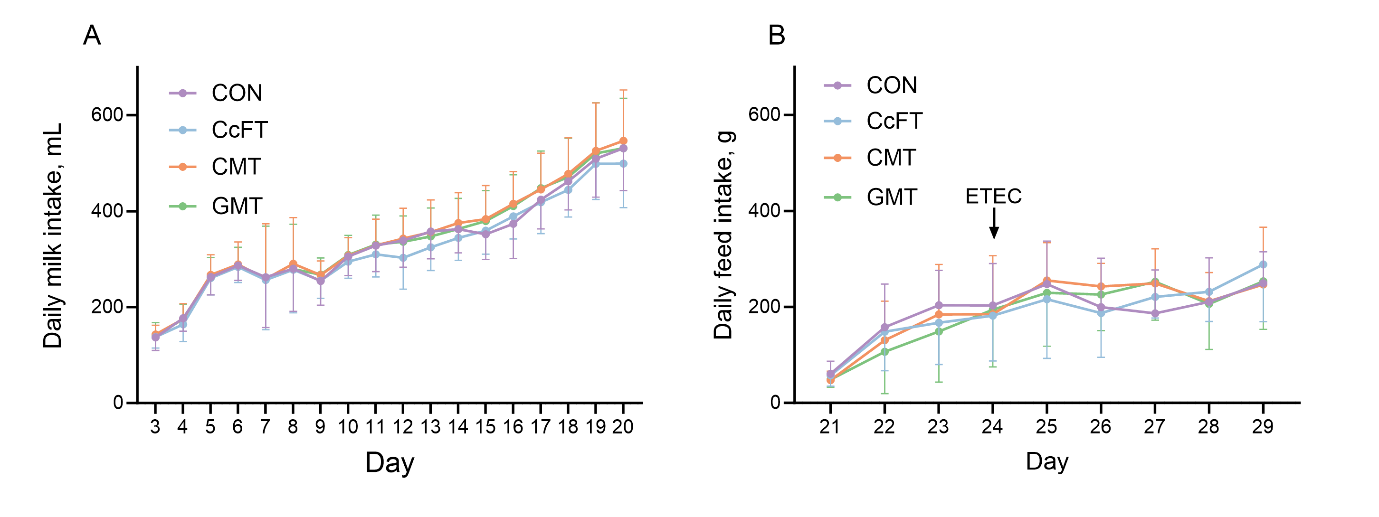
**

**Fig. S1** Feed intake

**
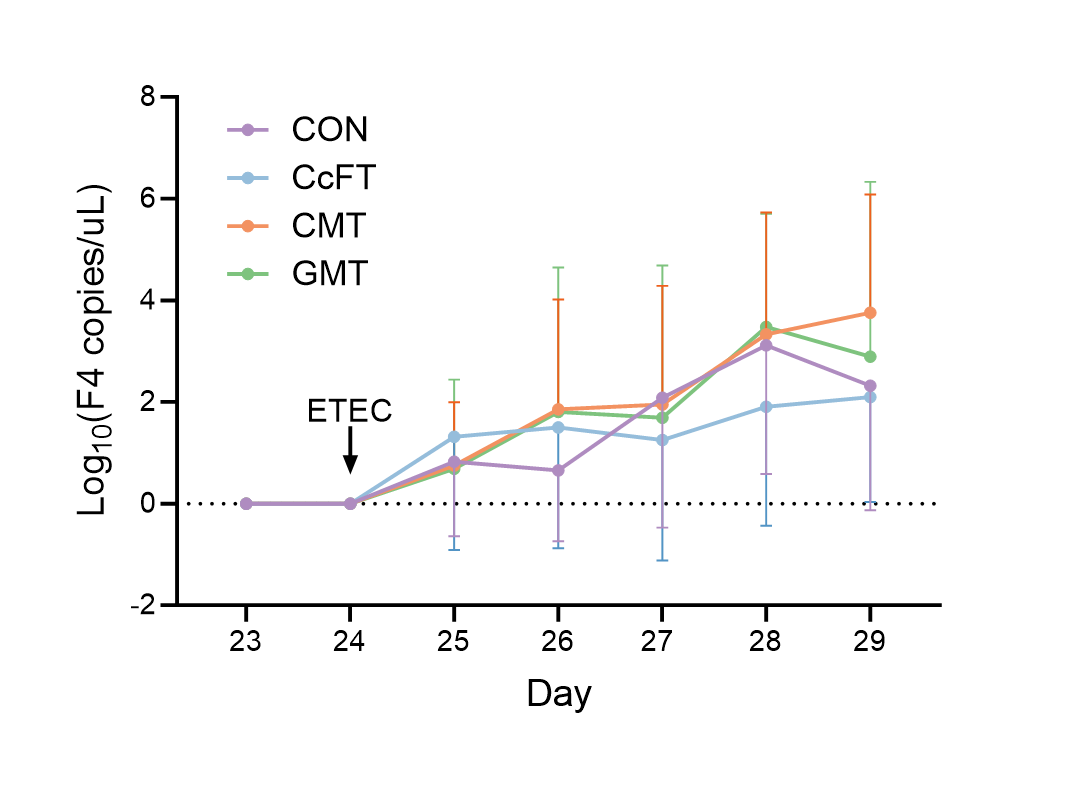
**

**Fig. 2** ETEC prevalence

**Table S1** Ingredients and calculated nutrient composition of the milk replacer provided to the piglets

| **Item** | **Content** |
| --- | --- |
| Bovine milk (DM), g/L^1^ | 135 |
| Bulk whey protein, g/L^2^ | 9.6 |
| Variolac 836, g/L^3^ | 19.2 |
| Lacprodan DI-9224, g/L^4^ | 9.6 |
| Nutrient composition |  |
| Energy, kJ/L | 3,907 |
| Protein, g/L | 53 |
| Whey protein, g/L | 24 |
| Carbohydrates, g/L | 65 |
| Sugars, g/L | 65 |
| Fat, g/L | 52 |

^1^Bovine milk from danish jersey cows

^2^Bulk whey protein (Bulk Powder Performance Delivered Pure Whey Protein, Bulk, Essex, United Kingdom) ^3^Variolac 836 (Arla Foods Ingredients P/S, Viby, DK)

^4^Lacprodan DI-9224 (Arla Foods Ingredients P/S)

All piglets received milk from d 2 of life and until d 21 of life

**Table S2** Ingredients and calculated nutrient composition of weaner^1^ diet

| **Item** | **Content** |
| --- | --- |
| Ingredients |  |
| Wheat, % | 53.7 |
| Barley, % | 10.0 |
| Fish meal, % | 5.0 |
| Hamlet Protein 300, % | 10.0 |
| Swine fat, % | 3.3 |
| B2B 25927 conc. 18 plus,% | 18.0 |
| Calculated netrient composition |  |
| DM, % | 88.8 |
| Crude protein % | 19.2 |
| Crude fat, % | 5.5 |
| Ash, % | 2.4 |
| Lactose, g/kg feed | 58.0 |

^1^All piglets received solid feed from d 22 to 29

**Table S3** Relative organ dimensions of piglets euthanized on d 29

| **Group** | **Unit** | **CON^1^** | **CcFT^2^** | **CMT^3^** | **GMT^4^** | ***P*-value** |
| --- | --- | --- | --- | --- | --- | --- |
| SI length | cm/kg | 243 ± 38.9 | 228 ± 26.8 | 232 ± 43.5 | 232 ± 44.7 | NS |
| SI proximal | g/kg | 20.3 ± 2.8 | 22.2 ± 6.3 | 19.0 ± 2.9 | 19.9 ± 2.4 | NS |
| SI medial | g/kg | 19.3 ± 2.5 | 20.1 ± 3.1 | 18.1 ± 2.4 | 18.7 ± 3.3 | NS |
| SI distal | g/kg | 21.0 ± 2.7 | 21.8 ± 3.0 | 20.9 ± 2.3 | 21.5 ± 2.1 | NS |
| Stomach cont. | g/kg | 82.1 ± 21.5**^a^** | 70.8 ± 17.2 | 57.9 ± 16.6**^b^** | 71.6 ± 24.6 | 0.01* |
| Stomach | g/kg | 12.6 ± 1.7 | 12.5 ± 1.8 | 12.2 ± 2.1 | 12.7 ± 1.8 | NS |
| Colon cont. | g/kg | 27.4 ± 7.9 | 27.6 ± 9.7 | 26.7 ± 7.6 | 23.9 ± 9.4 | NS |
| Colon | g/kg | 20.2 ± 05.4 | 19.1 ± 3.1 | 19.9 ± 5.9 | 19.0 ± 3.0 | NS |
| Liver | g/kg | 32.7 ± 7.4 | 35.6 ± 9.5 | 29.8 ± 6.4 | 28.8 ± 6.5 | NS |
| Spleen | g/kg | 2.3 ± 0.3**^aA^** | 2.9 ± 0.7**^B^** | 3.1 ± 1.0**^b^** | 2.6 ± 0.6 | 0.002* |
| Kidney | g/kg | 6.5 ± 0.7 | 6.6 ± 0.6 | 6.7 ± 0.8 | 6.2 ± 1.1 | NS |

^1^Control, CON; *n* = 16

^2^CcFT, Colonic content transplantation; *n*= 16

^3^CMT, Colonic microbiota transplantation; *n* = 15

^4^GMT, Gastric microbiota transplantation; *n* =15

**^a,b^**Values within a row with different superscripts differ significantly between treatments at *P* < 0.05

**^A,B^**Values within a row with different superscripts differ as a tendency between the treatments at *P* < 0.10

NS: Not significant. Values are expressed as mean ± SD. Organ dimensions are normalized to the body weight on d 29

**Table S4** Small intestine morphology of piglets on d 29

| **Group** | **CON^1^** | **CcFT^2^** | **CMT^3^** | **GMT^4^** | ***P*-value** |
| --- | --- | --- | --- | --- | --- |
| Height of villi, µm | 271 ± 48.9 | 281 ± 56.4 | 290 ± 65.3 | 254 ± 70.6 | NS |
| Depth of crypts, µm | 226 ± 25.6 | 223 ± 30.2 | 242 ± 26.0 | 229 ± 30.5 | NS |
| Height of enterocytes, µm | 28.7 ± 3.40 | 29.6 ± 3.85 | 28.7 ± 4.34 | 29.0 ± 4.71 | NS |
| Number of infiltration epithelium lymphocytes^5^ | 13.2 ± 3.14 | 13.5 ± 3.97 | 14.7 ± 4.15 | 13.3 ± 3.67 | NS |
| Number of goblet cells^5^ | 3.75 ± 2.52 | 4.20 ± 1.92 | 3.55 ± 1.92 | 4.03 ± 2.07 | NS |

^1^Control, CON; *n* = 16

^2^CcFT, Colonic content transplantation; *n* = 16

^3^CMT, Colonic microbiota transplantation; *n* = 15

^4^GMT, Gastric microbiota transplantation; *n* =15

^5^Per 100 enterocytes. Values are expressed as mean ± SD. NS: Not significant

**Table S5** Small intestine histopathology of piglets on d 29

| **Group** | **CON^1^** | **CcFT^2^** | **CMT^3^** | **GMT^4^** | ***P*-value** |
| --- | --- | --- | --- | --- | --- |
| Infiltration of the stromal mucosa | 0.50 (2.0) | 0.0 (1.0) | 1.0 (2.0) | 1.0 (1.0) | NS |
| Gut-associated lymphoid tissue | 0.0 (0.75) | 0.0 (10.75) | 0.0 (0.0) | 0.0 (0.0) | NS |
| Mucosal epithelium | 0.0 (0.0) | 0.0 (1.75) | 0.0 (2.0) | 0.0 (0.0) | NS |
| Brush border | 0.0 (1.0) | 0.0 (0.75) | 0.0 (2.0) | 0.0 (1.0) | NS |
| Intestinal blunting | 1.0 (0.75) | 1.0 (1.75) | 1.0 (1.0) | 1.0 (1.0) | NS |
| Cell detritus rich in eosinophils | 0.0 (0.0) | 0.0 (0.0) | 0.0 (0.0) | 0.0 (0.0) | NS |
| Erosion/ulcer | 0.0 (0.0) | 0.0 (0.0) | 0.0 (0.0) | 0.0 (0.0) | NS |
| Hyperplasia of enterocytes | 0.0 (0.0) | 0.0 (0.0) | 0.0 (0.0) | 0.0 (0.0) | NS |
| Oedema of stromal mucosa | 1.0 (1.0)**^A^** | 1.0 (1.0) | 0.0 (1.0) | 1.0 (1.0)**^B^** | 0.06 |
| Vessel dilation in stromal mucosa of villi | 0.0 (1.0) | 0.0 (0.75) | 0.0 (1.0) | 0.0 (1.0) | NS |
| Oedema of the submucosa | 2.0 (1.0) | 2.0 (1.0) | 2.0 (0.0) | 2.0 (0.0) | NS |
| Cell detritus on the villi surface | 0.0 (0.0) | 0.0 (0.0) | 0.0 (0.0) | 0.0 (0.0) | NS |
| Increase mitoses | 1.0 (2.0) | 1.0 (1.0) | 1.0 (3.0) | 0.0 (2.0) | NS |
| Hyperemia | 1.0 (1.0) | 0.0 (1.0) | 1.0 (2.0) | 1.0 (2.0) | NS |
| Vacuolisation of neurons | 2.0 (1.0) | 3.0 (1.0) | 2.0 (1.0) | 3.0 (1.0) | NS |

^1^Control, CON; *n* = 16

^2^CcFT, Colonic content transplantation; *n* = 16

^3^CMT, Colonic microbiota transplantation; *n* = 15

^4^GMT, Gastric microbiota transplantation; *n* =15

^A,B^values within a row with different superscripts differ as a tendency between the treatments at *P* < 0.10

Values are expressed as median and IQR. NS: Not significant. Histopathological lesions were assigned to a 5-point severity scale

**Table S6** Gut brush border enzymes of piglets on d 29

| **Group** | **Unit** | **CON^1^** | **CcFT^2^** | **CMT^3^** | **GMT^4^** | ***P*-value** |
| --- | --- | --- | --- | --- | --- | --- |
| Disaccharidases | | | | | | |
| Lactase | U/g | 1.47 ± 0.73 | 1.64 ± 0.96 | 1.84 ± 1.22 | 1.44 ± 0.19 | NS |
| Maltase | U/g | 7.86 ± 3.59 | 8.52 ± 5.18 | 9.29 ± 5.82 | 8.33 ± 3.98 | NS |
| Sucrase | U/g | 0.79 ± 0.36 | 1.09 ± 0.75 | 0.95 ± 0.60 | 0.81 ± 0.52 | NS |
| Peptidases | | | | | | |
| ApN^5^ | U/g | 5.59 ± 2.37 | 6.09 ± 2.50 | 5.23 ± 2.46 | 5.43 ± 2.25 | NS |
| ApA^6^ | U/g | 1.63 ± 0.87 | 1.48 ± 0.75 | 1.38 ± 0.95 | 1.34 ± 0.67 | NS |
| DPP IV^7^ | U/g | 2.13 ± 0.79 | 2.57 ± 1.16 | 2.33 ± 1.20 | 2.10 ± 0.95 | NS |

^1^Control, CON; *n* = 16

^2^CcFT, Colonic content transplantation; *n* = 16

^3^CMT, Colonic microbiota transplantation; *n* = 15

^4^GMT, Gastric microbiota transplantation; *n* =15

^5^ApN, aminopeptidase N

^6^ApA, Aminopeptidase A

^7^DPP IV, Dipeptidyl peptidase 4

Values are expressed as mean ± SD. NS: Not significant

**Table S7** Hematological parameters of piglets on d 29

| **Group** | **Unit** | **CON^1^** | **CcFT^2^** | **CMT^3^** | **GMT^4^** | ***P*-value** |
| --- | --- | --- | --- | --- | --- | --- |
| Total leucocytes | bill/L | 11.3 ± 3.03 | 10.6 ± 3.10 | 11.2 ± 3.48 | 11.2 ± 7.78 | NS |
| Total erythrocytes | trill/L | 5.05 ± 0.45 | 4.82 ± 0.58 | 5.15 ± 0.48 | 4.93 ± 0.66 | NS |
| Hemoglobin | mmol/L | 5.67 ± 0.50 | 5.48 ± 0.60 | 5.85 ± 0.49 | 5.65 ± 0.69 | NS |
| Hematocrit | L/L | 0.29 ± 0.03 | 0.28 ± 0.03 | 0.30 ± 0.02 | 0.29 ± 0.04 | NS |
| MCH^5^ | fmol | 1.13 ± 0.04 | 1.14 ± 0.05 | 1.14 ± 0.04 | 1.15 ± 0.07 | NS |
| MCHC^6^ | mmol/L | 19.6 ± 0.56 | 19.4 ± 0.55 | 19.6 ± 0.63 | 19.5 ± 0.52 | NS |
| Trombocytter | bill/L | 416 ± 141 | 411 ± 120 | 403 ± 188 | 365 ± 156 | NS |
| MPC^7^ | fL | 9.46 ± 1.60 | 9.06 ± 1.07 | 9.19 ± 1.22 | 10.3 ± 1.80 | NS |
| MCV^8^ | fL | 57.2 ± 2.31 | 58.9 ± 3.34 | 58.0 ± 2.09 | 58.7 ± 3.61 | NS |
| MPV^9^ | g/L | 236 ± 14.1 | 237 ± 20.0 | 235 ± 15.9 | 237 ± 16.0 | NS |
| Neutrophiles | % | 60.3 ± 5.64 | 60.8 ± 7.97 | 56.6 ± 14.0 | 59.5 ± 12.3 | NS |
| Lymphocytes | % | 34.6 ± 5.90 | 34.0 ± 7.44 | 39.0 ± 13.5 | 36.2 ± 11.6 | NS |
| Monocytes | % | 3.13 ± 1.64 | 3.27 ± 2.90 | 2.26 ± 1.02 | 2.23 ± 0.84 | NS |
| Eosinphils | % | 1.23 ± 1.04 | 1.12 ± 0.68 | 1.25 ± 1.77 | 1.23 ± 1.04 | NS |
| Basophils | % | 0.16 ± 0.09 | 0.13 ± 0.08 | 0.19 ± 0.24 | 0.12 ± 0.07 | NS |
| LUC^10^ | % | 0.53 ± 0.44 | 0.69 ± 0.45 | 0.67 ± 0.31 | 0.63 ± 0.38 | NS |
| Neutrophils | bill/L | 6.91 ± 2.30 | 6.61 ± 2.55 | 6.68 ± 2.78 | 7.28 ± 6.70 | NS |
| Lymphocytes | bill/L | 3.84 ± 0.94 | 3.53 ± 0.90 | 4.05 ± 1.28 | 3.65 ± 1.23 | NS |
| Monocytes | bill/L | 0.35 ± 0.16 | 0.33 ± 0.29 | 0.24 ± 0.12 | 0.24 ± 0.14 | NS |
| Eosinophils | bill/L | 0.14 ± 0.12 | 0.12 ± 0.07 | 0.15 ± 0.23 | 0.16 ± 0.18 | NS |
| Basophils | bill/L | 0.02 ± 0.01 | 0.02 ± 0.01 | 0.02 ± 0.01 | 0.01 ± 0.01 | NS |
| LUC^10^ | bill/L | 0.06 ± 0.04 | 0.07 ± 0.05 | 0.07 ± 0.03 | 0.07 ± 0.06 | NS |
| Reticulocytes | % | 2.28 ± 1.48 | 2.63 ± 1.61 | 2.57 ± 1.63 | 2.54 ± 1.71 | NS |
| Absolut reticulocytes | bill/L | 114 ± 71.2 | 121 ± 64.7 | 131 ± 79.2 | 120 ± 73.7 | NS |

^1^Control, CON; *n* = 16

^2^CcFT, Colonic content transplantation; *n* = 16

^3^CMT, Colonic microbiota transplantation; *n* = 15

^4^GMT, Gastric microbiota transplantation; *n* =15

^5^MCH, mean corpuscular haemoglobin

^6^MCHC, mean corpuscular haemoglobin concentration

^7^MPC, Mean platelet count

^8^MCV, Mean cell volume

^9^MPV, Mean platelet volume

^10^LUC, Large unstained cells

Values are expressed as mean ± SD. NS: Not significant

**Table S8** Biochemistry parameters of piglets on d 29

| **Group** | **Unit** | **CON^1^** | **CcFT^2^** | **CMT^3^** | **GMT^4^** | ***P*-value** |
| --- | --- | --- | --- | --- | --- | --- |
| Albumin | g/L | 26.1 ± 2.51 | 25.8 ± 2.80 | 26.3 ± 3.49 | 25.3 ± 3.23 | NS |
| Total protein | g/L | 42.0 ± 3.92 | 43.2 ± 3.05 | 43.7 ± 3.99 | 43.3 ± 4.62 | NS |
| Total bilirubin | umol/L | 0.20 ± 0.41 | 0.13 ± 0.34 | 0.64 ± 1.08 | 1.40 ± 1.64 | NS |
| Creatinine | umol/L | 58.8 ± 10.7 | 62.1 ± 17.0 | 60.8 ± 9.37 | 62.1 ± 15.5 | NS |
| Iron | umol/L | 29.5 ± 7.57 | 32.0 ± 14.4 | 26.1 ± 7.66 | 25.9 ± 11.8 | NS |
| AST^5^ | u/L | 52.7 ± 49.2 | 58.1 ± 32.8 | 47.1 ± 22.0 | 64.6 ± 53.1 | NS |
| Urea nitrogen | umol/l | 2.67 ± 2.36**^a^** | 2.86 ± 2.36 | 4.54 ± 3.16**^b^** | 4.66 ± 2.63 | 0.04 ^*#^ |
| GGT^6^ | u/L | 9.80 ± 8.43 | 10.6 ± 7.82 | 12.1 ± 7.49 | 9.86 ± 5.07 | NS |
| Calcium | mmol/L | 2.69 ± 0.15 | 2.79 ± 0.24 | 2.64 ± 0.19 | 2.53 ± 0.22 | NS |
| Magnesium | mmol/L | 0.92 ± 0.11 | 0.94 ± 0.0 | 0.89 ± 0.07 | 0.85 ± 0.09 | NS |
| Glucose | mmol/L | 6.90 ± 0.89 | 7.20 ± 1.66 | 6.30 ± 1.46 | 5.89 ± 1.61 | NS |
| CK^7^ | u/L | 302 ± 217 | 345 ± 250 | 283 ± 133 | 285 ± 215 | NS |

^1^Control, CON; *n* = 16

^2^CcFT, Colonic content transplantation; *n* = 16

^3^CMT, Colonic microbiota transplantation; *n* = 15

^4^GMT, Gastric microbiota transplantation; *n* =15

^5^AST, alanine aminotransferase

^6^GGT, gamma-glutamyl tranferase

^7^CK, creatine kinase

**^a,b^**Values within a row with different superscripts differ significantly between treatments at *P* < 0.05

NS: Not significant. Values are expressed as mean ± SD. ^*^*P* < 0.05. ^#^ log-transformed data used to fit model assumptions

**Table S9.** Health indices in blood serum of piglets on d 29

| **Group** | **Unit** | **CON^1^** | **CcFT^2^** | **CMT^3^** | **GMT^4^** | ***P*-value** |
| --- | --- | --- | --- | --- | --- | --- |
| IFNalpha | pg/mL | 6.07 ± 9.28 | 11.5 ± 23.1 | 51.5 ± 115 | 74.4 ± 190 | NS |
| IFNgamma | pg/mL | 0 ± 0 | 0 ± 0 | 0 ± 0 | 0 ± 0 | NS |
| IL-1beta | pg/mL | 0 ± 0 | 0.41± 1.69 | 0 ± 0 | 0 ± 0 | NS |
| IL-4 | pg/mL | 0.03 ± 0.13 | 0.02 ± 0.08 | 0.01 ± 0.03 | 0 ± 0 | NS |
| IL-6 | pg/mL | 0 ± 0 | 0 ± 0 | 0 ± 0 | 0 ± 0 | NS |
| IL-8 | pg/mL | 74.7 ± 85.3 | 49.4 ± 66.7 | 163 ± 189 | 175 ± 201 | NS |
| IL-10 | pg/mL | 3.19 ± 12.8 | 0 ± 0 | 0.14 ± 0.53 | 0 ± 0 | NS |
| TNFalpha | pg/mL | 1.47 ± 5.87 | 0 ± 0 | 0 ± 0 | 0 ± 0 | NS |
| IL-12p40 | pg/mL | 110 ± 147 | 126 ± 211 | 106 ± 140 | 204 ± 354 | NS |
| Haptoglobin | ug/mL | 566 ± 324 | 629 ± 597 | 474 ± 355 | 647 ± 577 | NS |
| CRP^5^ | ug/mL | 22.7 ± 13.6 | 53.8 ± 64.8 | 32.9 ± 22.5 | 41.7 ± 29.4 | NS |
| MAP^6^ | ug/mL | 51.5 ± 28.8 | 45.4 ± 17.6 | 42.9 ± 25.9 | 44.7 ± 31.6 | NS |

^1^Control, CON; *n* = 16

^2^CcFT, Colonic content transplantation; *n* = 16

^3^CMT, Colonic microbiota transplantation; *n* = 15

^4^GMT, Gastric microbiota transplantation; *n* =15

^5^CRP, C-reactive protein

^6^MAP, major acute phase protein

Values are expressed as mean ± SD. NS: Not significant
